# The use of new approach methodologies (high-throughput transcriptomics) to study nanoagrochemicals: mechanisms of toxicity of a commercial copper oxychloride to soil model invertebrates (*Enchytraeus crypticus*)

**DOI:** 10.1007/s00204-026-04304-3

**Published:** 2026-02-07

**Authors:** Susana I. L. Gomes, Janeck J. Scott-Fordsmand, Mónica J. B. Amorim

**Affiliations:** 1 Department of Biology & CESAM, University of Aveiro, 3810-193 Aveiro, Portugal; 2 Department of Ecoscience, Aarhus University, C.F. Møllers Alle 4, 8000 Aarhus, Denmark

**Keywords:** Precision agriculture, New approach methodologies (NAMs), Microarrays, Enchytraeids, Soil invertebrate, Non-target species, Econanotoxicology

## Abstract

Nanoagrochemicals aim to increase safety and sustainability, representing an alternative to conventional agrochemicals. Given the infancy of this field, the risk-benefit analysis for nano-enabled agrochemicals remains unresolved. New Approach Methodologies (NAMs), such as omics, are high on the agenda to move beyond standard hazards, as the investigation of the mechanisms of toxicity deliver valuable information to understand the risks. NUCOP–M^®^, a Cu-based commercial formulation containing nano-features, was previously assessed in *Enchytraeus crypticus* (Oligochaeta), impacting survival and reproduction based on the standard OECD Enchytraeid Reproduction Test (28 days) and its extension (56 days). The aim of the present study was to understand the mechanisms of toxicity of NUCOP–M^®^ at sublethal concentrations (100 and 500 mg Cu/kg soil of NUCOP-M^®^) in short and longer exposure (2, 21 days) using a high-throughput gene expression microarray (NAMs) containing probles for 44,000 genes (a 4 × 44 K microarray). Results showed high transcriptomic response after 21 days exposure, although after 2 days there was an indication of increase in intracellular transport. At 21 days, the gene expression profile was compatible with effects on embryo development, which could be linked to reproductive effects observed at 28- and 56-days of exposure. Several of the genes found up-regulated points towards mitochondrial stress and activation of mechanisms to cope with oxidative stress. This study provides important insights into the mechanisms of toxicity of nanoagrochemicals in non-target species, filling a significant knowledge gap.

**Graphical abstract:**

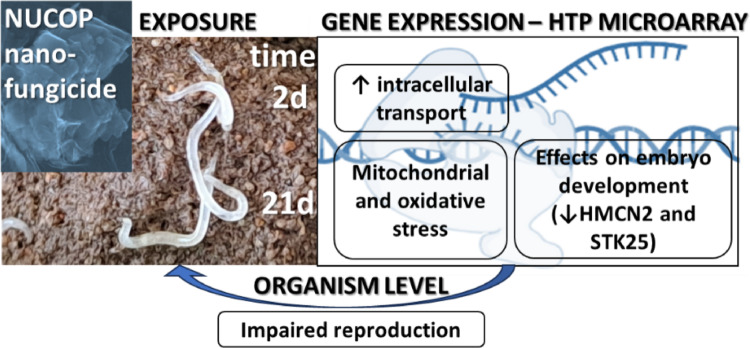

**Supplementary Information:**

The online version contains supplementary material available at 10.1007/s00204-026-04304-3.

## Introduction

Nano enabled agrochemicals (or nanoagrochemicals, i.e. chemical products used in agriculture that owe at least part of their activity to an inherent nanomaterial related property (Schwab 2023) aim to increase crop productivity without further increasing environmental pollution. There is potential for the use of nanotechnology to promote controlled and targeted release and delivery of the active substances or nutrients, improving efficiency (lower application rates) and reducing run-off residues (Pulizzi 2021). This tackles some of the major limitations of conventional agrochemicals (reduced application effectiveness and efficiency) and reduces the environmental footprint of agricultural practices, while contributing to addressing one of the major challenges of our time: to feed a growing population (Sharma et al. 2022; Ali et al. 2023).

Over the past few years, there has been a tremendous increase in research related to nanotechnology with agricultural applications, as illustrated by the number of patent applications and registrations, which almost doubled from 2011–2013 to 2014–2016 (Kah et al. 2019). Copper (Cu) has been used in agriculture as an antimicrobial agent for more than thirteen decades (Lamichhane et al. 2018). Thus, and considering the demand for more sustainable and eco-friendly crop pest control alternatives, it is no surprise that that Cu-based nanomaterials (Cu NMs) have been investigated for their potential use in agriculture (e.g. (Strayer-Scherer et al. 2018; Pariona et al. 2019; Oussou-Azo et al. 2020; Ahmad et al. 2020; Kohatsu et al. 2021; Ibarra-Laclette et al. 2022). Currently, some Cu-based nano enabled agrochemicals are already marketed, e.g. the fungicide Kocide^®^3000 (a copper hydroxide-based nano enabled formulation (Li et al. 2019). Toxicity studies with non-target species showed that Kocide^®^3000 induced mortality to the aquatic organisms *Daphnia magna* (96 h 50% Lethal Concentration: LC_50_ = 2.4 mg/L)(Aksakal and Arslan 2020) and zebrafish embryos (96hpf LC_50_ = 6.3 mg/L)(Aksakal and Sisman 2020) and that it was more toxic to zebrafish embryos than copper sulphate (Wang et al. 2021). Kocide^®^3000 and CuPRO 2005 (also a Cu-based pesticide) were as toxic to *Leptocheirus plumulosus* as CuCl_2_ (Vignardi et al. 2020). For soil living invertebrates, studies showed that Kocide^®^ 3000 was more toxic to *Folsomia candida* than the commercial non-nano formulations (Bordeaux mixture^®^, Cupravit^®^, and Nordox^®^) and as toxic as Champion^®^ (Neves et al. 2019). On the other hand, Kocide^®^ 3000 was least toxic to *Porcellionides pruinosus* and *Tenebrio molitor* in comparison to the other copper hydroxide-based pesticide (Champion^®^) and the pure active substance, and this was linked to a lower bioaccumulation potential (Morgado et al. 2022). Another commercial Cu-based nano enabled product (copper oxychloride based, NUCOP–M^®^) was less toxic in the short-term (2 and 13 days) than pure copper oxychloride and similarly toxic in the longer-term (28 and 56 days) to *Enchytraeus crypticus* (Chidiamassamba et al. 2024). A life cycle assessment (LCA) study to evaluate the environmental impacts of Cu-based nanopesticides in comparison to conventional pesticides (CuO Nps were used, in comparison to CuSO_4_) revealed that although conventional CuSO_4_ pesticides caused less environmental damage than CuO NPs (when per kg was used as the functional unit), CuO NPs consumed less renewable energy and presented better overall environmental performance (when per unit efficacy) (Liu et al. 2023). Another study estimated health risks of Cu-based nanoagrochemicals (Cu(OH)_2_ and CuO NPs), revealing that consumption of contaminated edible leafy vegetables resulted in health risks for humans. It also calculated maximum allowable applicable concentration values without posing risk to human health or causing plant toxicity to be 1.14 and 0.45 mg/L for Cu(OH)_2_ and CuO NPs, respectively (Shahane and Kumar 2022).

Despite the increasing data, the debate persists regarding the benefits of nanoagrochemicals in comparison to conventional products (i.e. regarding the efficiency gains and reduced toxicity (Zhang and Goss 2022; Paz-Trejo et al. 2023). The regulation of nanoagrochemicals needs further clarification for the adequate assessment of their safety (Grillo et al. 2021; Kah et al. 2021), especially understanding the nano forms’ influence on the pesticide related properties to assess exposure and fate. Hence, the understanding of the nanoagrochermical modes of action (or mechanisms of toxicity) will aid the current debate and clarify the comparative risks of nano versus conventional agrochemicals. Currently, the investigation of the modes of action of nanoagrochemicals is mostly focused on their target action (Schwab 2023), but the study of mechanisms of toxicity has also been recommended as part of the testing strategy for nanomaterials (Gomes et al. 2021) and nanopesticides (Grillo et al. 2021). Whitin an ecotoxicology perspective, a mechanistic based study showed that Kocide^®^3000 affects zebrafish larvae energy metabolism with decrease in glycolysis, activation of the adenosine monophosphate-activated protein kinase (AMPK)-mTOR signalling pathway, and promotion of TCA cycle (citrate cycle) (Wang et al. 2024). In *E. crypticus*, a microarray study indicated that an atrazine-based nanoformulation was absorbed by endocytosis, while pure atrazine is taken up by passive diffusion (Gomes et al. 2022), highlighting the differences between the nano and the non-nano pesticide.

The aim of the present study was to understand the mechanisms of toxicity of the commercial formulation NUCOP–M^®^, which has been reported to have nano-features.(Chidiamassamba et al. 2024) For that, a custom-made 4 × 44k high-throughput (HTP) microarray was used where 4 × 44,000 genes are printed on a microarray, representing its whole transcriptome (Castro-Ferreira et al. 2014; Gomes et al. 2017) to assess the transcriptomic profile of *Enchytraeus crypticus* (Oligochaeta) exposed to 100 and 500 mg Cu/kg of NUCOP–M^®^. These are known, sublethal concentrations, previously determined based on the standard OECD Enchytraeid Reproduction Test and its extension(Chidiamassamba et al. 2024) (28 days_reproduction 10% Effect Concentration: EC_10_ = 100 mg Cu/kg and 56 days_total organisms EC_50_ = 497 mg Cu/kg of NUCOP–M^®^). Enchytraeids are soil living invertebrates that have been used as test species in ecotoxicology studies for decades (Rombke 2003). They are non-target animals to agrochemicals exposure and a variety of tools exist to assess toxicity at different levels of biological organization, including the standard test to assess survival and reproduction(OECD 220 2016) and the HTP microarray(Gomes et al. 2017) for transcriptomic screening. The mechanisms of toxicity of the commercial fungicide NUCOP–M^®^, reported to have nano-features, are currently unknown.

## Materials and methods

### Test species

Adult animals of the species *Enchytraeus crypticus* (Westheide and Graefe, 1992) were used for the experiments. *E. crypticus* cultures were maintained in the laboratory for more than 10 years under controlled conditions of temperature (20 ± 2 °C) and photoperiod (16:8 h light: dark). The culture media consisted of sterilized Bacti-Agar medium (Oxoid, Agar No. 1) and a mixture of four different salt solutions at the final concentrations of 2 mM CaCl_2_⋅2H_2_O, 1 mM MgSO_4_, 0.08 mM KCl, and 0.75 mM NaHCO_2_. The cultures were fed with ground autoclaved oats twice a week.

### Test soil

The natural standard LUFA 2.2 soil (LUFA Speyer, Germany) was used as test media. The soils’ main characteristics were: pH (0.01 M CaCl_2_) = 5.5 ± 0.1; organic carbon = 1.72 ± 0.54%; cation exchange capacity (CEC) = 8.4 ± 1.9 meq/100 g; maximum water holding capacity (maxWHC) = 44.1 ± 6.0 g/100 g; grain size distribution = 10.7 ± 1.9% clay, 15.7 ± 1.1% silt, and 73.6 ± 2.1% sand.

### Test materials and spiking procedures

NUCOP–M^®^ 35% HiBIO^®^ (35% Cu w/w, in the form of copper oxychloride, Agrototal, Produtos Químicos S.A.), a commercially available copper-based fungicide, was tested as purchased. The fungicide formulation was previously characterized (Chidiamassamba et al. 2024) as a suspension containing irregularly shaped particles with a high degree of agglomeration (zeta average ranging from 876 to 1754 nm, with the presence of smaller peaks with ca. 300 nm).

The tested concentrations were 0, 100 and 500 mg Cu/kg soil dry weight. These concentrations cover the dose-response curve in terms of effects on reproduction at 28 and 56 days of exposure (28 days_reproduction EC_10_ = 100 mg Cu/kg; 56 days_total organisms EC_50_ = 497 mg/kg: ca. 50% effect concentration), as previously determined based on the standard OECD reproduction tests (28 days) and its extension for 56 days (Chidiamassamba et al. 2024).

NUCOP–M^®^ is commercialized as a water dispersible powder, thus it was added to soil as aqueous suspensions. A stock suspension was prepared and serially diluted with deionised water to obtain the test concentrations. The spiking followed the guidelines for nanomaterials (OECD 2012), with each replicate being prepared individually to ensure total raw amounts of the tested material. In short, the prepared suspensions were added to the pre-moistened soil to reach 50% of soil’s maxWHC, the soil was homogeneously mixed and left to equilibrate for 1 day prior to the start of the exposure.

### Exposure details

Exposure followed the standard OECD guideline for the Enchytraeid Reproduction Test (OECD 220 2016), with adaptations as described below. Forty adult animals with well-developed clitellum were introduced in each test vessel containing 20 g of moist soil (control or spiked) and 30 mg of food (ground autoclaved oats). The animals were exposed for 2 and 21 days under controlled conditions of photoperiod (16:8 h light: dark) and temperature (20 ± 1 °C). Food (33 mg) and water (based on weight loss) were replenished weekly. Four replicates per test condition and exposure period were performed. After respectively 2 and 21 days of exposure, the animals were carefully removed from the test soil, rinsed in deionized water and frozen in liquid Nitrogen. Samples were stored at − 80 °C till further analysis.

### Gene expression: microarray analysis

#### RNA extraction, labelling and hybridizations

Three out of the four biological replicates performed, consisting in a pool of 40 animals, were used for total RNA extraction. The 4^th^ replicate was only used for RNA extraction in the case of low-quality RNA. RNA was extracted using SV Total RNA Isolation System (Promega) according to the manufacturer’s protocol. RNA yield and purity were assessed spectrophotometrically using a NanoDrop instrument (ND-1000 Spectrophotometer), and its integrity was evaluated by denaturing formaldehyde agarose gel electrophoresis. A single-colour design was used. All the steps follow the Agilent kits manufacturers’ protocols, as previously described (e.g., in (Gomes et al. 2017, 2019, 2022, 2023). In brief, 500 ng of total RNA was amplified and labelled with Agilent Low Input Quick Amp Labelling Kit (Agilent Technologies, Palo Alto, CA, USA). Positive controls were added with the Agilent one-colour RNA Spike-In Kit (Agilent Technologies, Palo Alto, CA, USA). Purification of the amplified and labelled cRNA was performed with the RNeasy columns (Qiagen, Valencia, CA, USA). The cRNA samples were hybridized on the Custom Gene Expression Agilent Microarrays for this species (4 × 44k format (Gomes et al. 2017). Hybridizations were performed using the Agilent Gene Expression Hybridization Kit (Agilent Technologies, Palo Alto, CA, USA), and each biological replicate was individually hybridized on one array. The arrays were hybridized at 65 °C with a rotation of 10 rpm during 17 h. Following this, the microarrays were washed using Agilent Gene Expression Wash Buffer Kit (Agilent Technologies, Palo Alto, CA, USA) and scanned with the Agilent DNA microarray scanner G2505B (Agilent Technologies).

#### Acquisition and microarray data analysis

All procedures were performed in accordance with the manufacturers’ protocols and the data analysis pipeline, as previously described (e.g., in (Gomes et al. 2017, 2019, 2022, 2023). In brief, fluorescence intensity data was obtained with Agilent Feature Extraction Software v. 10.7.3.1 (Agilent Technologies). Quality control was done by inspecting the reports on the Agilent Spike-in control probes. Background correction was provided by Agilent Feature Extraction software v. 10.7.3.1, using the recommended protocol GE1_107_Sep09. To ensure an optimal comparison between the different normalization methods, only gene probes with good signal quality (flag IsPosAndSignif = True) in all samples were employed in the analyses. Analyses were performed with R (R 2015) v. 3.2.0, using the R packages plotrix and RColorBrewer and with Bioconductor (Huber et al. 2015) v. 3.3 packages genefilter and limma (Ritchie et al. 2015) v. 3.28.20. The data was normalized using the standard vector condition-decomposition method described in Roca et al. (2017). Differential expression between control and treated samples was assessed using linear models (limma) and Benjamini–Hoch- berg’s (BH) method to correct for multiple testing (Benjamini and Hochberg 1995) (adjusted *p* < 0.05 was considered significant). The Minimum Information About a Microarray Experiment (MIAME) compliant data from this experiment was submitted to the Gene Expression Omnibus (GEO) at the National Center for Biotechnology Information (NCBI) website (platform: GPL20310; series: GSE: GSE284246). The DEGs for each treatment were analyzed separately for GO (Gene Ontology) term enrichment analysis (Fisher’s exact test, *p* < 0.05) (Alexa et al. 2006) using the OmicsBox software (BioBam^®^, Bioinformatics Solutions). GO terms from the category biological processes were selected, as these relate to “biological functions”. The GO terms that were associated with only one transcript were removed from the significant GO term list. OmicsBox software was also used to produce the Veen diagram. Cluster analysis on differentially expressed genes (DEGs) was performed using MultiExperiment Viewer (MeV, TIGR) using Pearson’s uncentered correlation with average linkage. Principal Component Analysis (PCA) of samples was also performed using MultiExperiment Viewer (MeV, TIGR), based on the DEGs in at least one test condition and using the “Mean” centering mode. Annotation of microarray gene probes to the Kyoto Encyclopedia of Genes and Genomes (KEGG) (Kanehisa et al. 2016) was performed with the KEGG Automatic Annotation Server (KAAS) v. 2.1, (Moriya et al. 2007) using the representative set for eukaryotic species. Pathway expression analysis was done using Pathview Web, with pathway selection set to “auto” (Luo and Brouwer 2013). 

Following the Pathview methodology, the analysis took into account the expression ratios (treated versus control) of all the genes with annotation to KEGG orthologs.

## Results

**Fig. 1:**
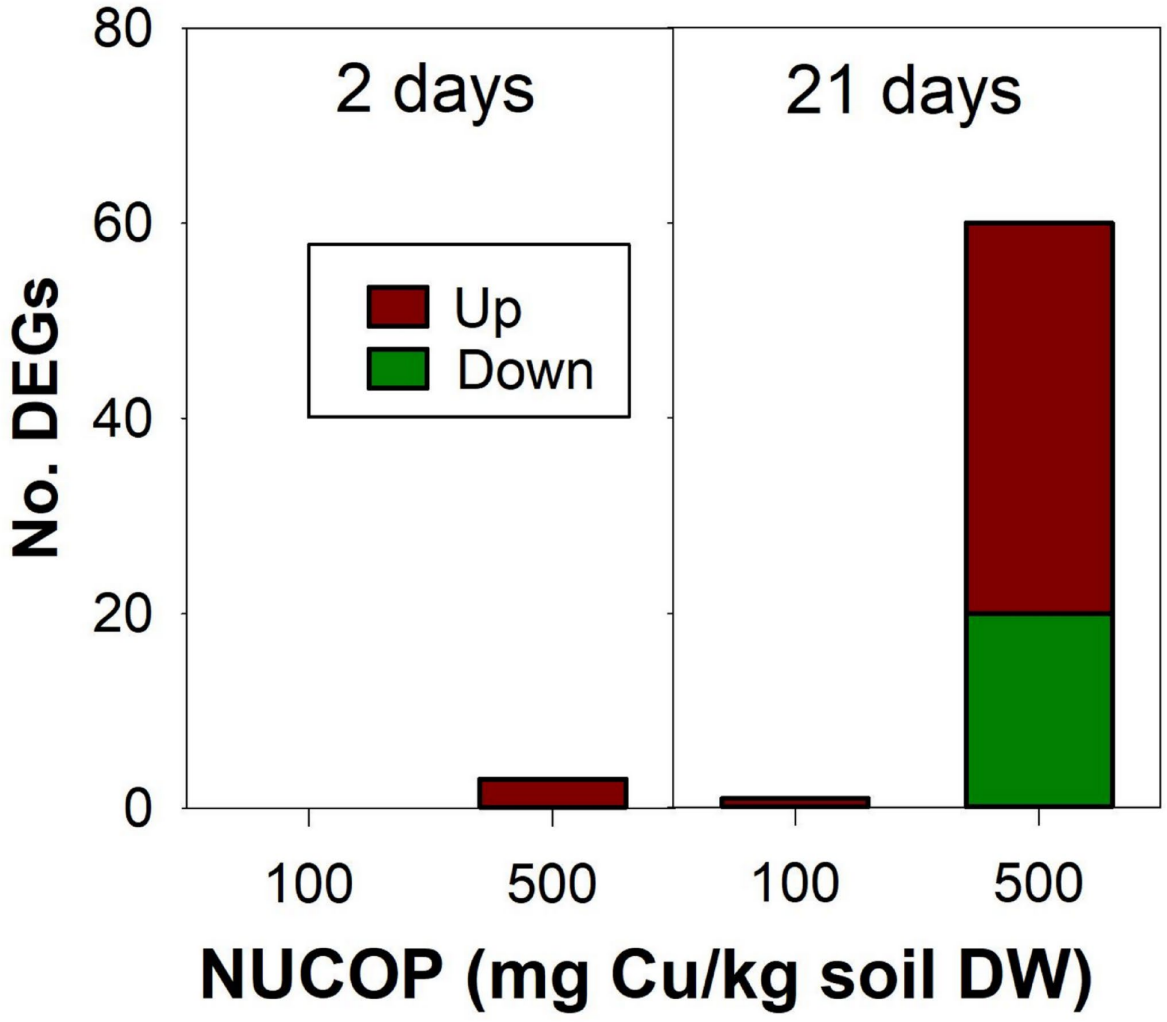
Results on the number of differentially expressed genes (DEGs) (adjusted *p* < 0.05) in *Enchytraeus crypticus* after exposure to NUCOP-M^®^ in LUFA 2.2 soil for 2 and 21 days. Up: up-regulated, and Down: down-regulated in comparison to the respective control

Among the 29,009 probes that passed the quality criteria, a total of 64 transcripts were found differentially expressed (adjusted *p* < 0.05) in at least one test condition. The vast majority of the differentially expressed genes (DEGs) was found for 500 mg/kg at 21 days of exposure, with no DEGs being found for 100 mg/kg at 2 days of exposure (Fig. 1).

The list of all the DEGs can be consulted in Table S1 (Supporting Information). There are no commonly affected genes, hence, no Venn diagram is presented.

**Fig. 2:**
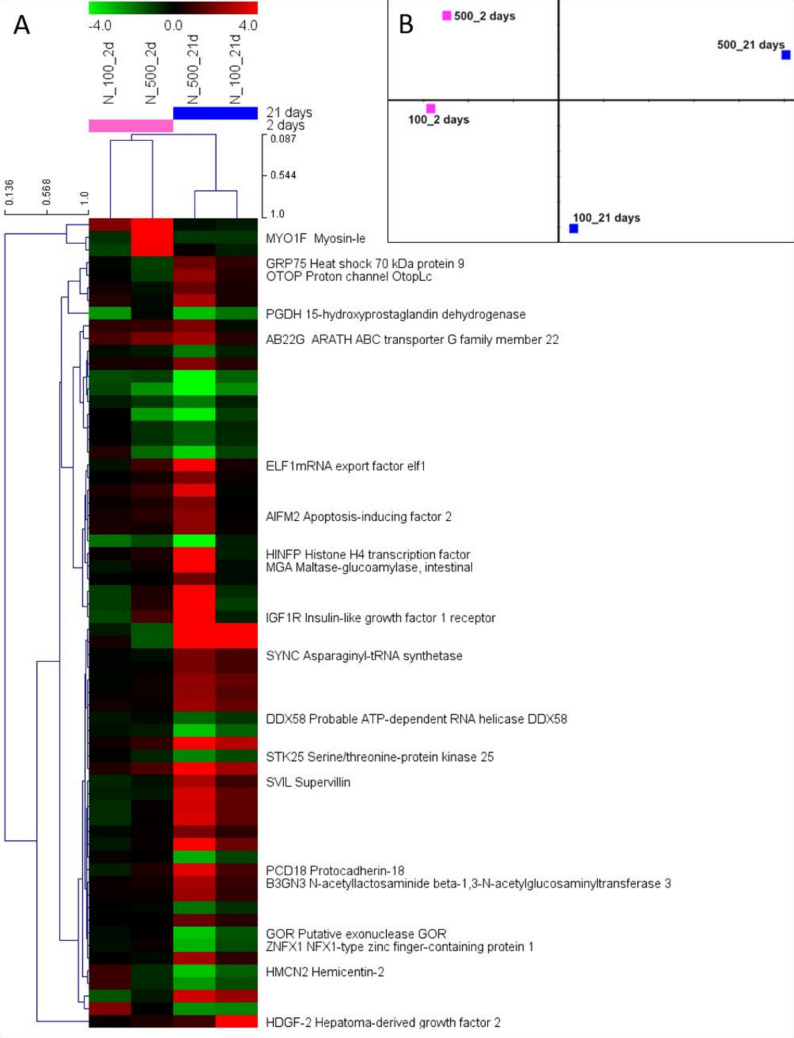
Result representation of all the differentially expressed genes (DEGs) (adjusted *p* < 0.05) in at least 1 test condition in *Enchytraeus crypticus* exposed to NUCOP–M^®^ at 100 and 500 mg Cu/kg, in LUFA 2.2 soil, for 2 and 21 days. (A) heat map of DEGs (log2 fold-change) and samples, hierarchically clustered using the Pearson uncentered correlation and average linkage, with the genes’ annotation included, and (B) principal component analysis (PCA) of samples [the first two components presented explain 92.803% of the data variance (PC1-x axis = 75.626%, PC2-y axis = 17.117%)]

The cluster analysis on genes and samples, done for the DEGs in at least one test condition and depicted in the heat map (Fig. 2A), show a clear separation by exposure time (the two times are separated at a similarity level of ca. 9%). Within each time, the samples (treatments) at 21 days are much more similar (ca. 65% similarity) than those at 2 days (ca. 10% similarity). Further, at 21 days there is a dose-dependent increase in the level of expression, i.e. more intense colours (red or green, indicating higher or lower expression) at 500 in comparison to 100 mg/kg. The separation of samples by exposure period is also visible in the PCA (Fig. 2B) across de x-axis, which explains 75.626% of data variance. However, in the PCA representation the treatments are closer at 2 days than at 21 days.

**Fig. 3:**
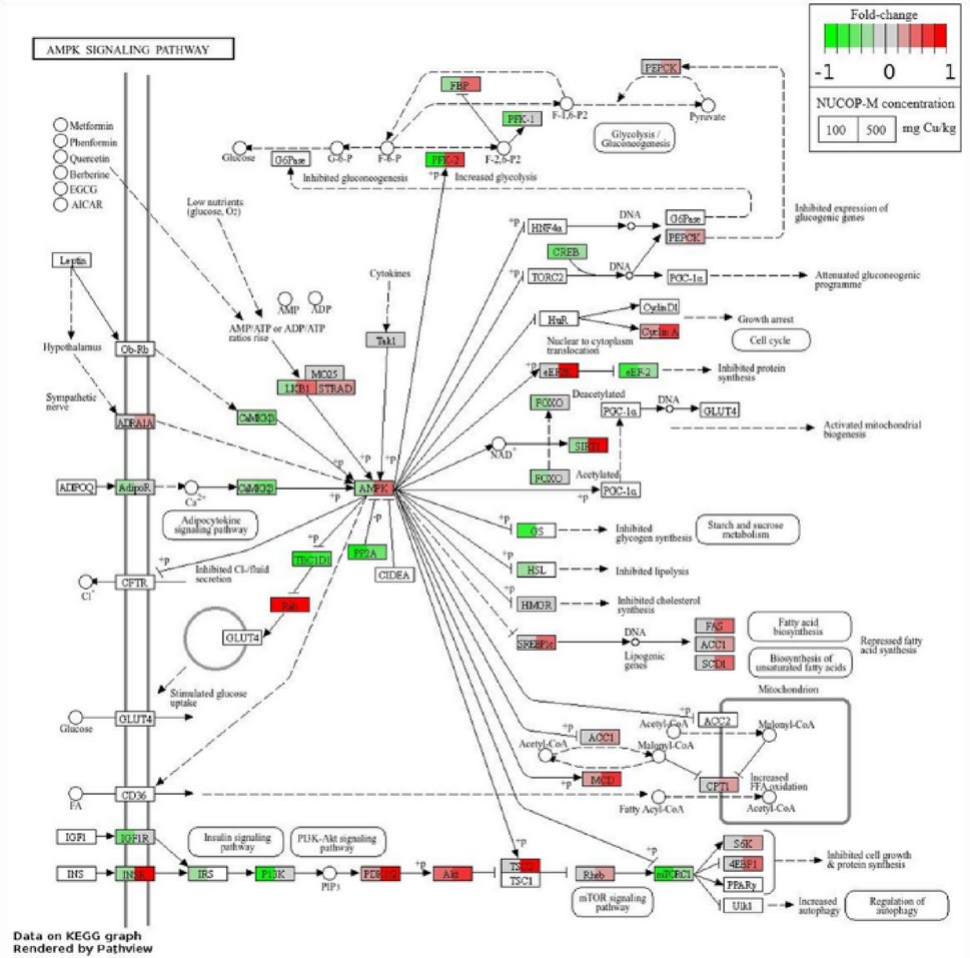
Fold-change (treatments versus control) of genes representing components of the “AMPK signalling pathway” KEGG pathway ko04152 as an example of pathways that are affected by exposure to NUCOP–M^®^ at 100 and 500 mg Cu/kg, for 21 days in *Enchytraeus crypticus*. Green and red indicate down- and up-regulation, respectively. Details in this pathway can be retrieved from the following website: https://www.genome.jp/kegg-bin/show_pathway?ko04152 (for interpretation of the references to colour in this figure legend, the reader is referred to the web version of this article)

No significant GO terms were found based on the criteria of enrichment analysis, thus, results will be discussed based on the DEGs. The pathway expression analysis was done for the two exposure periods separately due to the distinct gene expression patterns between days 2 and 21. No significant differentially expressed KEGG pathways (q-value < 0.2) were found. At day 21, the pathways ko04152 AMPK signalling pathway and ko00510 N-Glycan biosynthesis were the closest to being significantly affected (Fig. 3 and Fig. S1) (q-value = 0.2661).

## Discussion

NUCOP–M^®^ induced gene expression profiling is time and dose dependent, whereas minimal changes were triggered after 2 days, the higher concentration (500 mg/kg, ca. EC_50_ for total organisms_56 days) triggered an underlying high transcription. Several microarray studies have shown that gene expression is both highly time dependent and toxicant specific. For example, for *E. crypticus* exposed to nickel nanomaterial (Ni NM) there was a time and dose-dependent increase in the number of DEGs, from 3 to 7 days of exposure (Gomes et al. 2019). A previous study with *E. crypticus* exposed to Cu based materials showed a higher transcriptional response at 3 days (e.g. DEGs = 1109 for CuNPs) in comparison to 7 days (e.g. DEGs = 13 for CuNPs) for Cu NMs of different shapes (spheres and wires) (Gomes et al. 2018). Hence, we expected to capture a higher transcriptional response for NUCOP–M^®^ than what we actually did after 2 days exposure. It is unknown what is the exact reason for this difference, but CuNO_3_ also caused no DEG after 3-days of exposure (Gomes et al. 2018). The low response to NUCOP–M^®^ at 2 days could be due to an internal delayed NP effect or, perhaps, a reduced initial Cu exposure to NUCOP–M^®^. NUCOP-M^®^ is known to be designed to increase the release of Cu ions over time (https://agrototal.pt/produto/nucop-m-35-hibio/). We designed our study with the selection of 2 and 21 days of exposure to cover not only the early mechanisms of response to NUCOP–M^®^, but also responses that could be related to long(er)-term effects. Previous results indicate that Cu ions release would probably take more than 13 days to occur, at least up to a degree to induce toxicity at organism level (Chidiamassamba et al. 2024). Hence, although low, the gene expression profile of short-term exposure (2 days) should reflect the NUCOP-M^®^ (nano)formulation rather than Cu ions, which must be released later in time, probably the cause for the observed DEGs number at day 21. This discriminating gene expression profile of different toxicants/chemicals represents one of the major assets of toxicogenomic approaches (Hamadeh et al. 2002).

Moving towards specific observations, if we consider the most up-regulated genes, the unconventional class I myosin 1f (human MYO1F, homologous to) was uniquely affected at day 2. Unconventional myosins are essential in many biological processes, such as intracellular trafficking, mechanical support and force sensing, and have also been identified in *Caenorhabditis elegans* (although not specifically the corresponding to human ortholog MYO1F) (Johnson et al. 2022). Most of the studies on MYO1F are related to its functions on immune response (e.g. (Navinés-Ferrer et al. 2019; Martínez-Vargas et al. 2023). However, its role includes internalization and transport. For instance, primary macrophages lacking both myosin 1e and myosin 1f compromise the organization of individual adhesions, leading to excessive actin polymerization, slower adhesion turnover and deficient phagocytic internalization (Barger et al. 2019). Thus, it is possible that in *E. crypticus* MYO1F is involved in NUCOP–M^®^ particles internalization and/or transport inside the cells.

Among the genes down-regulated after 21 days of exposure to 500 mg Cu/Kg NUCOP–M^®^ were hemicentin 2 (HMCN2) and serine/threonine protein kinase 25 (STK25) [or the alternative name: Sterile 20/oxidant stress-response kinase 1 (SLK)]. Hemicentins are constituents of the extracellular matrix (ECM) from which proteins form networks that contain structural and regulatory information that influence cell adhesion, migration, survival, differentiation and polarity. Notably, its discovery in *C. elegans* was associated to defects in chromosome segregation with direct effects on animals’ germline and, thus, on reproduction (Vogel et al. 2006). STK25 is an oxidant stress-activated serine/threonine kinase involved in several cellular processes, including regulation of cell migration, modulation of cell death and energy metabolism (Nerstedt et al. 2012). Several studies show that the inhibition of STK25 (or SLK) by knock down experiments results in delayed cell death, cell cycle arrest and inhibition of cell migration, highlighting its role in several biological processes (reviewed by Al-Zahrani et al. (2013). Among the processes affected by STK25 (or SLK) is also embryo development. It has been shown that mice embryos that were SLK-deficient presented marked developmental defects leading to lethality, and one of the causes was reduced cell proliferation (Al-Zahrani et al. 2014). Considering the literature data, it is possible that the down-regulation of both hemicentin 2 and serine/threonine protein kinase 25 coding genes, occurring in *E. crypticus* exposed to 500 mg Cu/kg NUCOP–M^®^, contributed to the severe effects observed in terms of reproduction (ca. 90% reduction at 28 days, and 50% reduction at 56 days (Chidiamassamba et al. 2024).

The list of genes that were up-regulated by 500 mg Cu/kg NUCOP–M^®^ included those coding for: glucose-regulated protein 75 (GRP75), insulin-like growth factor I (IGF-1) and apoptosis-inducing factor 2 (AIFM2) [also known as ferroptosis suppressor protein 1 (FSP1)]. GRP75 is a mitochondrial matrix protein that regulates endoplasmic reticulum (ER)-mitochondrial Ca^2+^ transfer, with major roles on regulating mitochondrial function and redox homeostasis (Honrath et al. 2017). Researchers (Honrath et al. 2017) used immortalized neuronal HT22 cells as model and glutamate as oxidative stress causing agent. They showed that depletion of GRP75 provided protection against oxidative glutamate toxicity, while GRP75 overexpressed cells were more vulnerable to cell death and had increased sensitivity to oxidative stress through an increase in ER–mitochondrial contact formation (Honrath et al. 2017). In vivo and in vitro studies with ducks (Peng et al. 2023) and duck renal tubular epithelial cells (Wang et al. 2022) showed that copper exposure (CuSO_4_) caused mitochondrial dysfunction and ER stress by affecting mitochondria-associated ER membrane (MAM) structure. In both studies, GRP75 mRNA and protein levels were increased (Wang et al. 2022; Peng et al. 2023). Since NUCOP–M^®^ releases Cu ions over time (as also indicated by the producer https://agrototal.pt/produto/nucop-m-35-hibio/), it is likely that either Cu ions and/or the composed NUCOP–M^®^ formulation are inducing mitochondrial stress to *E. crypticus*. To counteract the mitochondrial stress, animals might have responded with the up-regulation of IGF-1, which has been implicated in preserving mitochondrial function (Hao et al. 2011; Ribeiro et al. 2014). However, considering the latter effects at organism level (i.e., reduction in reproduction (Chidiamassamba et al. 2024), the up-regulation of IGF-1 was not enough to prevent further damage and/or other mechanisms are involved. Ferroptosis is an iron-dependent regulated cell death mechanism driven by the induction of oxidative stress and accumulation of lipid peroxidation that can be triggered by copper excess (Aschner et al. 2022; Xue et al. 2023). The liver of chickens exposed to copper (CuSO_4_) was damaged by ferroptosis, and among other ferroptosis-related indicators was the down regulation of FSP1 (Zhong et al. 2024). The up-regulation of AIFM2 (or FSP1) in enchytraeids exposed to NUCOP–M^®^ indicates the activation of protective mechanisms against reactive oxygen species (ROS), as it inhibits lethal peroxidation and ferroptosis (Hadian 2020).

Taken together, our results indicate that enchytraeids are facing (and responding) to oxidative stress. Copper is known to induce oxidative stress and damage (e.g. (Pereira et al. 2016), as also studied in detail in enchytraeids (Gomes et al. 2012, 2014, 2018). One study on a Cu(OH)_2_ nanopesticide showed the increase in the mRNA levels of several antioxidant and detoxification-related genes in exposed cucumber plants (Zhao et al. 2017). A similar response was reported for CuSO_4_ exposed plants, and the authors (Zhao et al. 2017) concluded that released copper ions were triggering the oxidative stress response. Transcriptomics studies in *E. crypticus* exposed to various Cu materials (CuNO_3_, Cu salt aged, CuNPs, Cu nano wires) (Gomes et al. 2018) showed an overall decrease in DEGs from 3 to 7 days, except for salt aged Cu. This is not comparable to the longer 21 days exposure and, of course, these materials were designed for a different purpose. Nevertheless, it does seem to indicate that higher transcriptomic effects in the long-term (21d) exposure to NUCOP–M^®^ are associated with the observed effect in the animals’ decreased performance (decreased reproduction), as measure at day 28 and 46 (Chidiamassamba et al. 2024).

Among the few studies on the mechanisms of toxicity of nanoagrochemicals (Wang et al. 2024), it was shown that the Cu (hydroxide) based Kocide^®^3000 affected the energy metabolism of zebrafish larvae, with decrease in glycolysis and activation of the adenosine monophosphate-activated protein kinase (AMPK)-mTOR signalling pathway. In *E. crypticus* exposed to NUCOP–M^®^, the KEGG “AMPK signalling pathway” was affected. Interestingly, the inspection of the proteins involved (Fig. 2) indicated changes in expression towards cell cycle arrest, which is in line with the previous discussion. Further, energy metabolism might also be affected, but for NUCOP–M^®^ it seems to be towards the increase of glycolysis. Human hepatocellular carcinoma (HepG2) cell line exposed to Cu (hydroxide) nanoagrochemical exhibited a metabolomic profile associated with oxidative stress in which a shift from oxidative phosphorylation to glycolysis and lipid accumulation was observed (Li et al. 2022). This seems to be also the case for *E. crypticus* exposed to NUCOP–M^®^.

We realise over years of experience that even with specific exposure levels, i.e. EC_10_ or EC_50_, for different tested compounds, this induces different underlying transcriptions, including the intensity, scale and direction (up or down-regulation). The meaning is yet to be translated, e.g. whether there is a pattern within the findings and how it could be interpreted. Hence, it seems that even if the same ECx is being compared within different studies, it does not mean that the long-term impact will become comparable.

## Conclusions

The present study revealed possible mechanisms of toxicity of a commercial Cu oxychloride based nanoformulation to a soil invertebrate non-target species, which was completely absent from the literature. Although the higher transcriptional response was observed after 21 days of exposure in the current test-design, it was possible to detect an increase in cell transport after 2 days. NUCOP–M^®^ seems to directly affect embryo development at gene level. Much of the transcriptional response identified is related to induction and response to oxidate stress associated with mitochondria, as previously described for copper. Moreover, the current corroborates the longer-term impact observed at the organism level.

## Supplementary Information

Below is the link to the electronic supplementary material.

Supplementary Material 1

Supplementary Material 2

## Data Availability

Microarray data is available via Gene Expression Omnibus (GEO) at the National Center for Biotechnology Information (NCBI) website (platform: GPL20310; series: GSE: GSE284246). Further data will be made available on request.
