# Supplementary material for: The use of new approach methodologies (high-throughput transcriptomics) to study nanoagrochemicals: mechanisms of toxicity of a commercial copper oxychloride to soil model invertebrates (Enchytraeus crypticus)
Source: Arch Toxicol. 2026 Feb 7;100(5):1825–35. doi: 10.1007/s00204-026-04304-3 (PMC13086727; doi:10.1007/s00204-026-04304-3)
Supplement: Supplementary file 1 — Supplementary Material 1 [file 204_2026_4304_MOESM1_ESM.docx]

**Supplementary Information (SI)**

**The use of New Approach Methodologies (high-throughput transcriptomics) to study nanoagrochemicals - mechanisms of toxicity of a commercial copper oxychloride to soil model invertebrates (*Enchytraeus crypticus*)**

Susana I.L. Gomes^a^, Janeck J. Scott-Fordsmand^b^, Mónica J.B. Amorim^a,*^

^a^Department of Biology & CESAM, University of Aveiro, 3810-193 Aveiro, Portugal

^b^Department of Ecoscience, Aarhus University, C.F. Møllers Alle 4, DK-8000, Aarhus, Denmark

*corresponding author: [mjamorim@ua.pt](mailto:mjamorim@ua.pt)


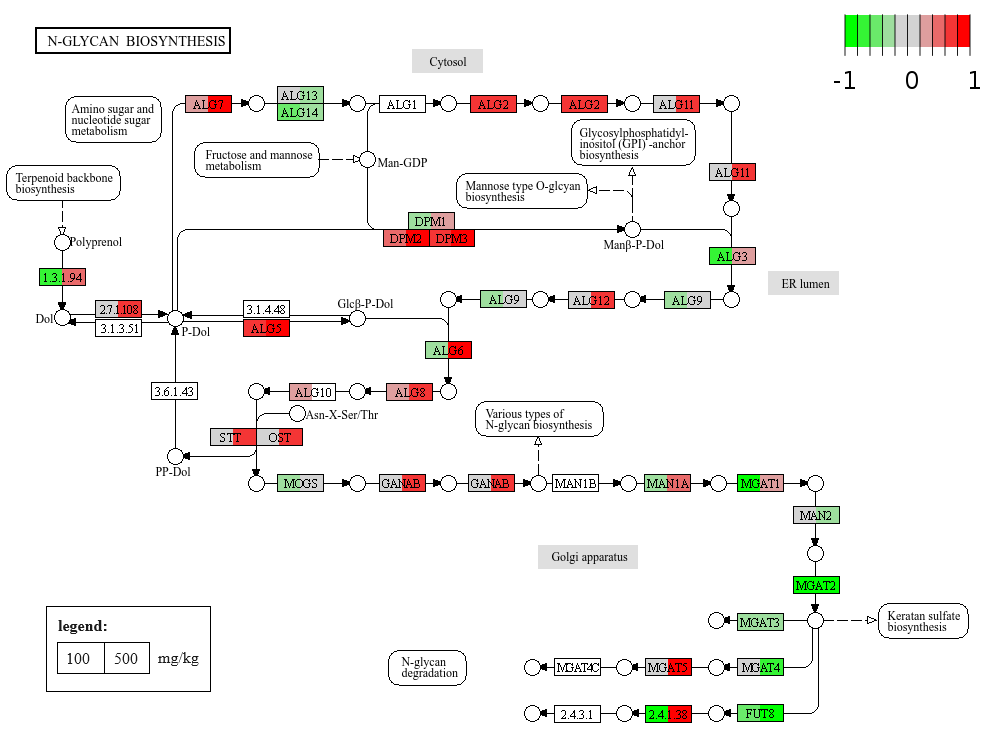


**Figure S1:** Fold-change (treatments versus control) of genes representing components of the “N-Glycan Biosynthesis” KEGG pathway ko00510, affected in *Enchytraeus crypticus* exposed to NUCOP-M® for 21 days, as rendered by Pathview. Green and red indicate down- and up-regulation, respectively. Details in this pathway can be retrieved from the following website: <https://www.genome.jp/pathway/ko>00510 (for interpretation of the references to colour in this figure legend, the reader is referred to the web version of this article).
